# Characterization of Proteins Involved in Chloroplast Targeting Disturbed by Rice Stripe Virus by Novel Protoplast–Chloroplast Proteomics

**DOI:** 10.3390/ijms20020253

**Published:** 2019-01-10

**Authors:** Jinping Zhao, Jingjing Xu, Binghua Chen, Weijun Cui, Zhongjing Zhou, Xijiao Song, Zhuo Chen, Hongying Zheng, Lin Lin, Jiejun Peng, Yuwen Lu, Zhiping Deng, Jianping Chen, Fei Yan

**Affiliations:** 1 The State Key Laboratory Breeding Base for Sustainable Control of Pest and Disease, Key Laboratory of Biotechnology in Plant Protection of MOA of China and Zhejiang Province, Institute of Virology and Biotechnology, Zhejiang Academy of Agricultural Sciences, Hangzhou 310021, China; 13297003755@163.com (J.X.); hzchenbinghua@aliyun.com (B.C.); cuiweijun0609@163.com (W.C.); zj_20020101@163.com (Z.Z.); songxijiao@yeah.net (X.S.); gychenzhuo@aliyun.com (Z.C.); zhenghongyinghz@163.com (H.Z.); linsnowx@163.com (L.L.); pengjiejun@yeah.net (J.P.); luyuwen@yeah.net (Y.L.); zhipingdeng@126.com (Z.D.); jpchen2001@126.com (J.C.); 2 Texas A&M University AgriLife Research Center at Dallas, Dallas, TX 75252, USA; 3 School of Biotechnology, Jiangnan University, Wuxi 214122, China; 4 Institute of Plant Virology, Ningbo University, Ningbo 315211, China; 5 Center of Research and Development of Fine Chemicals, Guizhou University, Guiyang 550025, China

**Keywords:** chloroplast proteomics, rice stripe virus, nuclear/nucleus-encoded chloroplast-related protein, plant–virus interaction, chloroplast targeting

## Abstract

Rice stripe virus (RSV) is one of the most devastating viral pathogens in rice and can also cause the general chlorosis symptom in *Nicotiana benthamiana* plants. The chloroplast changes associated with chlorosis symptom suggest that RSV interrupts normal chloroplast functions. Although the change of proteins of the whole cell or inside the chloroplast in response to RSV infection have been revealed by proteomics, the mechanisms resulted in chloroplast-related symptoms and the crucial factors remain to be elucidated. RSV infection caused the malformation of chloroplast structure and a global reduction of chloroplast membrane protein complexes in *N. benthamiana* plants. Here, both the protoplast proteome and the chloroplast proteome were acquired simultaneously upon RSV infection, and the proteins in each fraction were analyzed. In the protoplasts, 1128 proteins were identified, among which 494 proteins presented significant changes during RSV; meanwhile, 659 proteins were identified from the chloroplasts, and 279 of these chloroplast proteins presented significant change. According to the label-free LC–MS/MS data, 66 nucleus-encoded chloroplast-related proteins (ChRPs), which only reduced in chloroplast but not in the whole protoplast, were identified, indicating that these nuclear-encoded ChRPswere not transported to chloroplasts during RSV infection. Gene ontology (GO) enrichment analysis confirmed that RSV infection changed the biological process of protein targeting to chloroplast, where 3 crucial ChRPs (K4CSN4, K4CR23, and K4BXN9) were involved in the regulation of protein targeting into chloroplast. In addition to these 3 proteins, 41 among the 63 candidate proteins were characterized to have chloroplast transit peptides. These results indicated that RSV infection changed the biological process of protein targeting into chloroplast and the location of ChRPs through crucial protein factors, which illuminated a new layer of RSV–host interaction that might contribute to the symptom development.

## 1. Introduction

### 1.1. Plant Virus and Chloroplast

Plant viruses are obligatory intracellular parasites and normally encode relatively fewer proteins compared to other types of pathogens. Thus, the propagation and spreading of plant viruses are exclusively dependent on host cellular machinery and metabolism [1,2,3,4]. Viral infections usually cause plant chlorosis, stunting, necrosis, or other symptoms [5,6]. Leaf chlorosis, being the most common viral symptom, implies reduced photosynthetic activity of chloroplasts [7,8]. In addition, the multiple changes associated with the symptoms of chlorosis, such as reduced chlorophyll pigmentation [9,10], changes in chloroplast structures and functions [11,12], repressed expression of nuclear-encoded chloroplast photosynthesis-related genes (*ChRGs*) [5,13,14], as well as metabolism accumulation of nitric oxide [15], suggest essential interactions between virus and the chloroplast [16]. 

Many studies indicated that virus infection always caused changes in chloroplast structure, components, and expression of *ChRGs*, which is assumed to be responsible for the development of viral symptoms [17,18,19,20,21,22]. For instance, there were chloroplasts with stroma vacuolization, rearrangement of the thylakoids, and cytoplasmic invagination in barley leaf cells infected with barley stripe mosaic virus (BSMV) [23]. The helper component-proteinase (HC-Pro) of potato virus Y (PVY) can interact with the chloroplast ATP synthase NtCF1β-subunit, leading to a decrease of ATP synthase complex in both the HC-Pro transgenic and the PVY-infected tobacco, suggesting that HC-Pro is involved in reducing the photosynthetic rate of PVY-infected plants [24]. The tobacco mosaic virus (TMV) coat protein (CP) can interact with the chloroplast ferredoxin I (Fd I) [25]. The chlorotic phenotype and the accumulation of the TMV CPs in the chloroplasts were enhanced by the decrease of Fd I in TMV-infected tobacco leaves, suggesting that the interaction of CP and Fd I may contribute to the occurrence of chlorosis and mosaic symptoms [26].

Nowadays, increasing studies revealed that the chloroplast, as well as its factors, can interact with or be the targets of viral components or factors to favor the replication, movement, and symptom development of viruses [16,27]. For example, the PsbP (photosystem II oxygen-evolving complex protein) interacts with alfalfa mosaic virus (AMV) CP, and its overexpression observably hindered virus replication [28]. The RuBisCO small subunit could be hijacked by the tobamovirus movement proteins to facilitate the intercellular movement of virus [29,30]. The 3′-UTR region of bamboo mosaic virus (BaMV) interacts with chloroplast phosphoglycerate kinase (CpGK). Silencing or mislocalization of the cPGK gene in *N. benthamiana* reduced BaMV accumulation [31]. Alternanthera mosaic virus (AltMV) RNA interference (RNAi) suppressor TGB1_L88_ specifically binds to the chloroplast β-ATP*ase* (CF1β). Silencing of *CF1β* gene induced severe tissue necrosis in AltMV-infected *N. benthamiana* plants, suggesting that the interaction of CF1β and TGB1_L88_ weakened severe symptoms caused by AltMV infection [32]. These studies implied that the interactions of chloroplasts and plant viruses are commonly related to the formation of chlorosis symptoms during virus infection.

### 1.2. RSV Infection and Chloroplast Proteomics 

RSV is one of the most devastating rice pathogens causing dramatic losses in rice production in East Asia [33,34]. RSV is the typical member of the genus *Tenuivirus* and is transmitted by the small brown plant-hopper (*Laodelphax striatellus* Fallén) in a persistent and circulative-propagative manner [35]. RSV-infected rice leaves initially appear white or contain yellow spots, which develop into white or yellow stripes, then the RSV-infected leaves become yellow, with necrotic areas magnified from the stripes and, finally, lead to death of the whole leaf [33,36]. It was suggested that the chlorosis symptom was caused by disruption of normal chloroplast components and functions [17,18,22]. For instance, the downregulation of *ChRG* genes in RSV-infected rice plants is assumed to be responsible for chlorosis of RSV symptoms [6]. Quantitative proteomics analysis of rice leaves reveal that the accumulation of 30 ChRPs was apparently reduced by RSV infection [37]. However, how the localization and function of these ChRPs were modulated during RSV infection remains obscure. So far, there is no special methodology to reveal how the localization change of the nucleus-encoded ChRP factors is affected by RSV infection at the proteomic level. In recent years, mass spectrometry-based proteomics has developed rapidly as a useful approach for the identification of all the host proteins that play roles in virus infections [17,38]. Label-free quantitative proteomics technique is growing in popularity due to faster, cleaner, and simpler mass spectrometry results with higher technical reproducibility [38,39]. In this study, we used a label-free quantitative proteomics approach to analyze protein accumulation profiles of protoplast and chloroplast from RSV-infected leaves. By combining the quantified protoplast and chloroplast proteomic data, we established a method to elucidate the localization change of nucleus-encoded ChRPs and indicate a new layer of RSV–host interaction where the targeting of nucleus-encoded ChRPs is hindered during RSV infection.

### 1.3. RSV Infection of Nicotiana benthamiana Plants

In nature, RSV mainly infects rice plants and many other different species of plants in the family Poaceae, including wheat (*Triticum aestivum* L.) and maize (*Zea mays* L.) [40,41]. RSV-infected rice plants exhibit common features, such as chlorotic and necrotic stripes in leaves [42]. The chlorotic and necrotic stripes appear on a few areas of the leaves at first, then the chlorotic areas enlarge and show necrosis along leaf veins, and eventually lead to plant wilting [6,43]. The development of viral symptoms suggested that the RSV infection affects the mesophyll cells unevenly in different areas in rice leaves. Therefore, rice leaves with heterogeneous symptoms of RSV infection are not suitable for explaining the general effects of the virus on chloroplast proteins, or for isolating chloroplasts and protoplasts affected by virus infection. As a susceptible host, *N. benthamiana* could be systemically infected by RSV and showed symptoms of unanimous chlorosis [44], which makes *N. benthamiana* suitable for isolating chloroplasts and protoplasts affected by virus infection and explaining the general effects on chloroplast proteins caused by RSV infection. Thus, we used *N. benthamiana* as the host for RSV infection to provide insights into the influence on nucleus-encoded ChRP proteins. 

## 2. Results

### 2.1. The Phenotype and the Ultrastructure Change of Chloroplasts in RSV-Infected N. benthamiana Plants 

**Figure 1:**
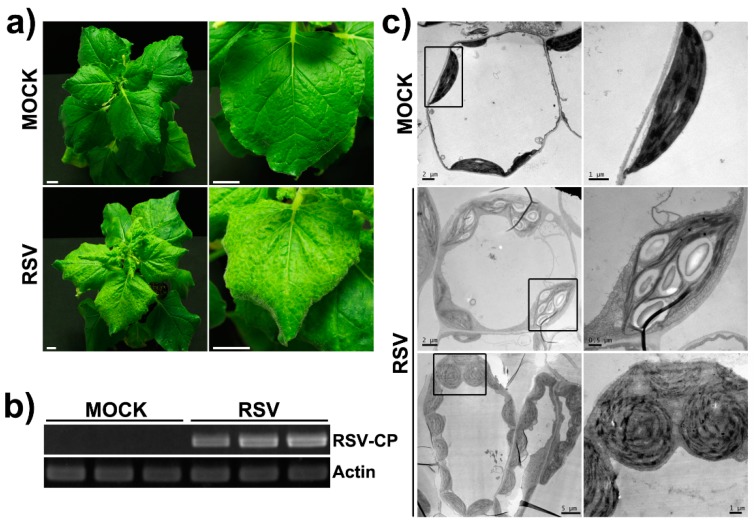
Influence of rice stripe virus (RSV) infection on *N. benthamiana* plants and the ultrastructure of chloroplasts from *N. benthamiana* leaves. (**a**) Phenotype of RSV-infected *N. benthamiana* plants at 25 dpi. (**b**) Viral RNA accumulation in mock plants and RSV-infected plants were analyzed by RT-PCR. (**c**) Ultrastructure of chloroplasts from RSV-infected *N. benthamiana* plants. Right panels are the magnified images of the line-boxed area in the left panels, respectively. MOCK, the mock-inoculated healthy *N. benthamiana* plants; RSV, the RSV-infected *N. benthamiana* plants.

*N. benthamiana* plants infected with RSV began to show mild yellowing symptoms in systemic leaves at 12–15 days post-inoculation (dpi). At 20–25 dpi, RSV-infected systemic leaves exhibited distinct symptoms such as backward curling, yellow vein, and unanimous chlorosis in the whole leaf area (Figure 1a). Systemic spreading of virus was confirmed by reverse transcription PCR (RT-PCR) using specific primers to the *CP* gene of RSV (Figure 1b). The yellowing symptoms indicated the abnormality of the chloroplast in *N. benthamiana* leaf cells. By electron microscopy, the ultrastructure of chloroplasts in RSV-infected *N. benthamiana* plants revealed dramatic malformations. There were swollen or globular chloroplasts, enlarged and over-accumulated starch grains inside chloroplasts, and a reduction or irregular arrangement of stroma lamella (Figure 1c).

In addition, we examined the ultrastructure of chloroplast in RSV-infected *Oryza sativa* plants. The ultrastructure of the chloroplasts from RSV-infected rice leaves also became abnormal, such as enlarged starch grains, exaggeration of electron-dense granules, disappearance of grana stacks, altered grana with dilated thylakoids scattering into the cytoplasm, appearance of an inter-membranous sac, and broken chloroplast envelopes with disrupted membrane (Supplemental Figure S1b). RT-PCR showed that RSV was accumulated in the symptomatic leaves of the RSV-infected *O. sativa* plants (Supplemental Figure S1a). These results suggest that the structure of chloroplasts became abnormal in RSV-infected plant leaves.

### 2.2. Blue Native PAGE (BN-PAGE) of Chloroplast Membrane Complexes of RSV-Infected Plants

**Figure 2:**
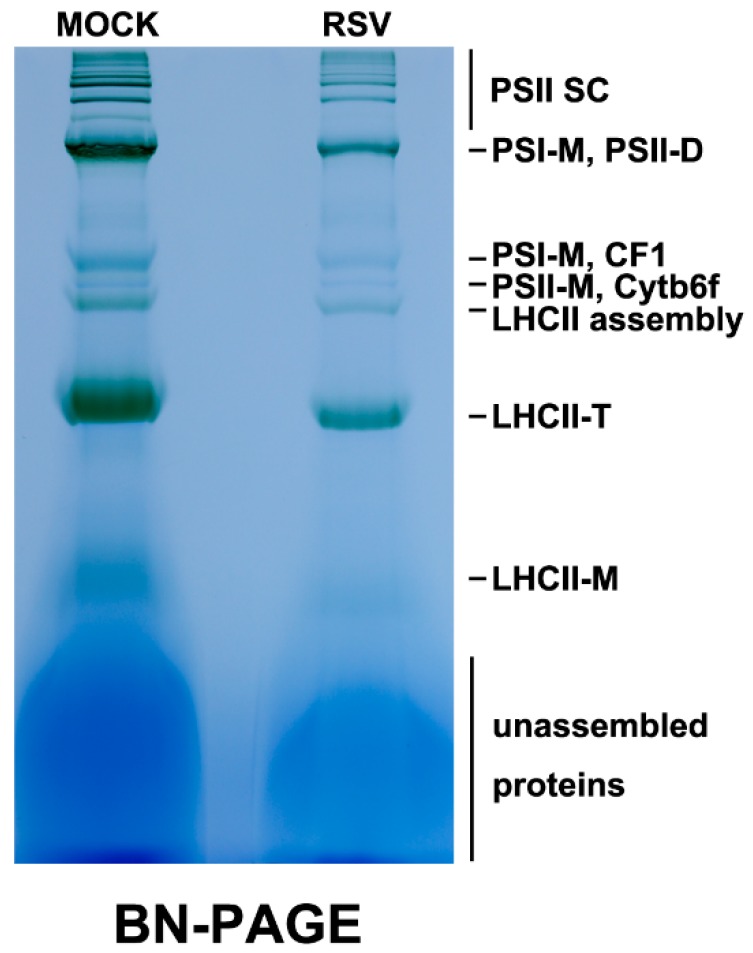
Blue native PAGE of chloroplast membrane protein complexes isolated from an equal quantity of healthy leaves (MOCK) and RSV-infected leaves (RSV). MOCK, the mock-inoculated healthy *N. benthamiana* leaves; RSV, the RSV-infected *N. benthamiana* leaves. PSII-SC, PSII supercomplex; PSI-M, PSI monomer; PSII-D, PSII dimer; LHCII, PSII light-harvesting complex; LHCII-T, PSII light-harvesting complex trimer; LHCII-M, PSII light-harvesting complex monomer; Cytb6f, cytochrome b6/f complexes.

Since the structure of the chloroplasts in RSV-infected *N. benthamiana* leaves became abnormal, we investigated the components and the quantity of the chloroplast membrane protein complexes in RSV-infected plants. The protein complexes of chloroplast membrane isolated from healthy leaves (MOCK) and RSV-infected leaves (RSV) were analyzed by blue native PAGE (Figure 2). As illustrated in Figure 2, a global reduction of the chloroplast membrane proteins was observed after RSV infection. PSI-M and PSII-D significantly decreased the most among these complexes. These results indicated that RSV infection reduced the abundance and the constitution of the chloroplast membrane protein complexes in *N. benthamiana* plants, which implied that the whole constitution of the chloroplast proteins and their routine functions were highly perturbed due to RSV infection. Thus, it is assumed that RSV infection might impose influence on the transport of chloroplast proteins. 

### 2.3. Western Blot Analysis of the Specificity of Chloroplast Protein Extracts

**Figure 3:**
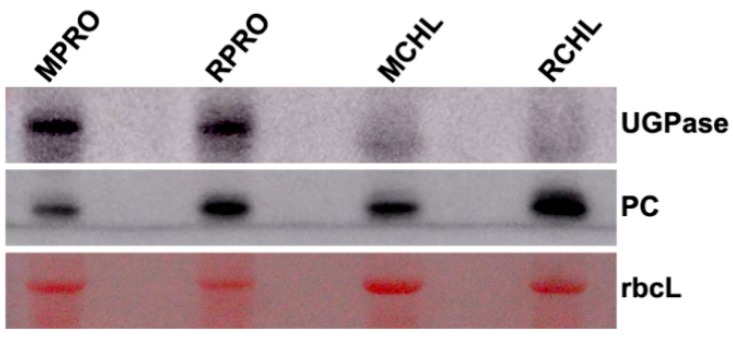
Analysis of the specificity of the chloroplast fraction by Western blot. MPRO, protein extracts of protoplasts from mock-inoculated healthy leaves; RPRO, protein extracts of protoplasts from RSV-infected leaves; MCHL, protein extracts of chloroplasts isolated from protoplasts from mock-inoculated healthy leaves; RCHL, protein extracts of chloroplasts isolated from protoplasts from RSV-infected leaves. UGPase, UDP-glucose pyrophosphorylase (cytoplasm marker); PC, plastocyanin (chloroplast marker); rbcL, RuBisCO large subunit.

In order to investigate the nuclear-encoded ChRPs, whose process of transporting into chloroplast was influenced by RSV infection, we established a novel protoplast–chloroplast proteomic approach to screen them. The proteins of protoplast and chloroplast were extracted from mock-inoculated healthy leaves and RSV-infected *N. benthamiana* leaves, respectively. In order to assess the specificity of the chloroplast fraction, the marker proteins of protoplast and chloroplast were detected by WB. UDP-glucose pyrophosphorylase (UGPase) and plastocyanin (PC) were used as cytoplasmic and chloroplast markers, respectively. The WB results showed that the fraction of chloroplast did not contain UGPase, which was a specific marker in cytoplasm (Figure 3), suggesting that the chloroplast fraction did not contain cytoplasmic components. Then, the proteins extracted from the protoplasts and chloroplasts were analyzed by label-free LC–MS/MS, in hopes of finding the nucleus-encoded ChRPs, to which the process of transporting to chloroplasts was hindered by RSV infection.

### 2.4. Analysis of Protoplast and Chloroplast Proteomics by Label-Free LC–MS/MS

Total proteins were extracted from three independent biological replicates of protoplasts and chloroplasts from either the mock-inoculated healthy or the RSV-infected leaves and subjected to nano LC–MS/MS (Supplemental Figure S2). The changes of proteins in chloroplasts and protoplasts were investigated by comparative proteomic analysis combined with label-free quantification. A total of 5640 MS/MS spectra were obtained from the protoplast samples by LC–MS/MS. The acquired MS/MS spectra were subsequently analyzed by Spectronaut 10 and searched against the tomato UniProt Fasta database, the RSV proteins, and the Biognosys iRT peptide sequences, where 4668 unique peptides were assigned (Supplemental Table S1) and represented 1128 identified proteins (Supplemental Table S2). The average value of median CVs from healthy and RSV-infected protoplast samples were 19.9% and 16.2%, respectively. Totally, 1128 proteins identified from protoplast samples were taken for protein abundance comparisons between RSV-infected and the mock-inoculated control *N. benthamiana* leaf samples. A total of 494 proteins showed a significant difference in abundance (*p* < 0.05, Supplemental Table S3), and a heat map of these proteins was obtained with a fold change (FC) of at least ±0.58 (Supplemental Figure S3). A ratio of protein abundance value above 2, in RSV-infected protoplast samples compared to that of the mock-inoculated protoplast samples (RPRO/MPRO), indicated an increase in abundance, 0.5–2 indicated no abundance change, less than 0.5 indicated a decrease in abundance.

In terms of the chloroplast samples, there were 3699 MS/MS spectra and 2876 unique peptides (Supplemental Table S4), representing 659 identified proteins (Supplemental Table S5). The average value of median CVs from the mock-inoculated healthy chloroplast samples and the RSV-infected chloroplast samples were 27% and 51.5%, respectively. A total of 279 chloroplast proteins showed significant difference in abundance upon RSV infection (*p* < 0.05, Supplemental Table S6), and a heat map of these proteins was obtained with an FC of at least ±0.58 (Supplemental Figure S4). The ratio of the abundance in value of RCHL/MCHL above 2 indicates increased accumulation, 0.5–2 indicates little change, and less than 0.5 indicates decreased accumulation.

To further confirm the quality of the sample collection, we examined the accumulation of PC, UGPase, and fibrillarin as markers for chloroplast, protoplast, and nucleus, respectively, in the identified proteins from protoplast and chloroplast samples. It was found that PC existed in both protoplast and chloroplast samples, and UGPase and fibrillarin only existed in the protoplast. It was obvious that the chloroplast fractions were not contaminated by cytoplasm and nuclear components, which was consistent with previous WB results.

### 2.5. GO Enrichment Analysis of the Identified Candidate Proteins

Based on the protoplast–chloroplast proteomics, a selection filter, where the abundance of candidates only decreased in the RSV-infected chloroplast samples but not in RSV-infected protoplast samples, was used to screen the nucleus-encoded ChRPs which did not transport into chloroplasts during RSV infection. The chloroplast targeting of the candidate proteins were reconfirmed in UniProt, *N. benthamiana* genome, and *Arabidopsis* Chloroplast Target gene databases [45,46,47,48,49]. A total number of 66 nucleus-encoded ChRPs which did not transport into chloroplasts after RSV infection were obtained, as shown in Table 1.

The 66 candidate proteins were subjected to BLAST against the Solanaceae genome database to gain the gene ID of the 66 proteins in Solanaceae. Gene ID of the 66 candidate proteins were submitted to gene ontology (GO) enrichment analysis using GOEAST. It was shown that 48 genes among the 66 genes have GO annotation (Supplemental Figure S5). It is worth noting that several pathways relevant to protein targeting to the chloroplast were enriched in the “biological process” category. This result indicated that RSV infection affected the pathways of protein targeting to the chloroplast. This is consistent with our proposal of the altered location of ChRPs during RSV infection, which can be validated by the protoplast–chloroplast proteomics method. Thus, this protoplast–chloroplast proteomics method successfully identifies the perturbed translocation of chloroplast proteins after RSV infection. This is the first identification of chloroplast proteins that do not transport into chloroplasts during RSV infection. 

From the GO terms, three nuclear-encoded ChRPs (K4CSN4, K4CR23, and K4BXN9, Table 2) were enriched, representing the enriched chloroplast-targeting biological process (Supplemental Figure S5). This result implied that RSV infection blocked the normal localization and function of these three proteins from importing the substrate factors into the chloroplast which, in turn, lead to the abnormal location of more chloroplast proteins. These results suggest that RSV infection changed the routine biological process of protein targeting into chloroplast and, thus, resulted in the abnormal location of chloroplast proteins and may contribute to the abnormal structure and function of the chloroplasts.

### 2.6. Transit Peptide Analysis of the Candidate ChRPs

In addition to the above three key proteins (K4CSN4, K4CR23, and K4BXN9), the function of the other 63 candidate proteins remains to be further analyzed for their involvement in chloroplast function and relation to viral symptom development. The chloroplast transit peptide (TP) is a leader sequence that directs the chloroplast protein import into the chloroplast, making its presence crucial for the localization of the protein to the chloroplast. We analyzed and confirmed, by TargetP 1.1 (Supplemental Table S7), that these three proteins had this transit peptide. Therefore, we speculated that the three proteins might regulate the localization of chloroplast proteins by their present in the chloroplasts. In addition, according to the transit peptide analysis by TargetP 1.1 [50], the majority (65%) of the 63 candidate proteins were found to have the chloroplast transit peptide (Supplemental Table S7), which further confirmed that the candidate proteins are ChRPs with abnormal location. 

GO enrichment analysis of these 41 candidate proteins with chloroplast transit peptide (Supplemental Figure S6) revealed that the associated biological pathways of protein targeting to the chloroplast, represented by the same three proteins (K4CSN4, K4CR23, and K4BXN9), were further enriched (Supplemental Figure S6), suggesting that the candidate proteins with predicted chloroplast transit peptide are more dependent on the biological process of protein targeting into the chloroplast. Thus, these three proteins play an important role in the localization of chloroplast proteins. 

## 3. Discussions

### 3.1. The Coincidence of the Chloroplast Perturbation with the RSV Symptoms 

Previous studies have indicated that the viral symptom of chlorosis was associated with the changes of chloroplast structure [16,20,51,52]. For instance, the severe chlorosis induced by CMV-P6 infection was basically related to the reduction in the number of the chloroplasts as well as the grana in chloroplasts [53]. Distorted thylakoid membranes may promote the shorter chlorophyll fluorescence lifetime of chlorotic leaves from plant virus-infected plant [54].

Some studies have also indicated that changes in chloroplast structure were often accompanied by changes in the chloroplast membrane protein complexes during plant infection. For instance, the TMV *flavum* strain infection caused a complete depletion of the monomeric and dimeric PSII complexes accompanied with chlorotic symptoms in tobacco plants [55], which is similar to our results revealed by BN-PAGE. In addition, TMV CP could embed into or form a complex with thylakoid membranes. The chloroplasts from tissues with more severe mosaic symptoms tended to accumulate more TMV CPs, up to 10–50 fold than those from symptomless leaves, thus indicating that TMV CP associated with thylakoid membranes may play a possible role in symptom development [56]. The co-localization of a tobacco protein IP-L and ToMV CP in the chloroplast thylakoid membranes suggested the interaction between the tobamovirus CPs and thylakoid membrane components, which may affect the function and stability of chloroplasts, thus leading to chlorosis [57]. These studies revealed that plant virus infection caused chlorosis-related symptoms which were coincident with the changes of chloroplast membrane complexes and chloroplast structure.

Many studies indicated that RSV infection always caused changes in chloroplast components, structure, and ChRPs in varied layers. For example, the downregulation of *ChRGs* in RSV-infected rice plants (*Oryza sativa* L.) is assumed to be responsible for the RSV chlorosis symptoms [6]. RSV disease- specific protein (SP) interacts with the ChRP 23 kDa oxygen evolving complex (OEC) PsbP, and resulted in the downregulation of PsbP in chloroplasts, which could modulate RSV symptoms through disruption of chloroplast structure and function [58]. In a recent report, iTRAQ-based quantitative proteomic analysis of rice leaves revealed that the accumulation of 30 proteins related to chloroplasts was apparently reduced by RSV infection [37]. The above studies suggested RSV infection have a tremendous influence on chloroplast structure. In this study, we found that the *N. benthamiana* infected by RSV exhibited tremendous changes of chloroplast ultrastructure and unanimous chlorosis lamina and yellow vein as well. It is suggested that the chloroplast ultrastructure changes and phenotypic changes were induced simultaneously in RSV-infected plants.

### 3.2. Targeting of ChRPs into Chloroplast is an Essential Layer of Virus–Chloroplast Interaction 

The fully functioning process of ChRPs contains a series of steps. The plant virus can impose their effects on varied layers, such as transcription, post-transcription, translation, targeting into chloroplast, complex assembly, and degradation, thus contributing to virulence and related symptoms [16,55,59,60]. Plant virus protein factors, such as TMV mutant CPs which can aggregate outside of chloroplasts, may subvert chloroplast development and cause degradation of chloroplasts by interfering with the synthesis and transport of CPRPs [16,55,59,60,61,62,63]. Several research sources have reported viral proteins disturbing ChRPs from their normal transportation into chloroplasts. Tobamovirus MPs can interact with the ChRP RuBisCO small subunit (RbCS) at plasmodesma (PD), which contribute to cell-to-cell movement, as well as the systemic transport of TMV and ToMV [30]. The sugarcane mosaic virus (SCMV) HC-Pro protein specifically interacts with ChRP ferredoxin V (Fd V) via the transit peptide, and probably perturbs the importing of Fd V into chloroplasts from outside of the chloroplasts [22]. As mentioned above, RSV SP interacts with PsbP, and relocates it from the chloroplast into the cytoplasm, which is associated with the enhanced RSV symptom [58].

Here, we isolated the chloroplasts from the protoplasts and validated the specificity by WB detection of marker proteins. Then, the chloroplast proteomic and protoplast proteomic profiles were analyzed by label-free LC–MS/MS, and specific filters were set to obtain the 66 nuclear-encoded ChRPs which were not targeted into chloroplast after RSV infection. As revealed by GO enrichment analysis and chloroplast transit peptides analysis, most of these candidate proteins were associated with the biological process of protein targeting into the chloroplast. It is worth noting that several of the candidate ChRPs have also been characterized as described [37], such as P37222 (NADP-dependent malic enzyme), K4CNE8 (ATP-dependent Clp protease proteolytic subunit), and Q0MW94 (1-deoxy-d-xylulose-5-phosphate reductoisomerase). With this unique quantified chloroplast–protoplast proteomics, we demonstrated that chloroplast importation of ChRPs is an essential layer for RSV to manipulate the routine processes of plant hosts during the plant–virus interaction, which is related to virulence and symptom development.

### 3.3. Application of Chloroplast–Protoplast Proteomics

A growing number of studies have shown that viral proteins can associate with or transport into various organelles, and lead to malfunction or stress response of the organelles [64,65,66,67]. For instance, RSV p2 could recruit fibrillarin in the nucleus and promote virus systemic infection by manipulating nuclear functions [68]. The groundnut rosette virus (GRV) ORF3 protein enters into the nucleus by targeting and reorganizing the Cajal bodies (CBs) into multiple CB-like structures that can fuse with the nucleolus [69]. The tomato bushy stunt virus (TBSV) 33 kDa replication protein (p33) was sorted into peroxisome and endoplasmic reticulum (ER), which induced altered peroxisome rearrangements [70]. The two closely related tombusvirus carnation Italian ringspot virus (CIRV) and melon necrotic spot carmovirus (MNSV) lead to mitochondrial damage by inducing multivesicular bodies (MVBs) from the outer membrane of mitochondria [71,72,73]. Several lipid membrane-associated virus factors, such as PVX TGBp3 [74,75], rice black-streak dwarf virus (RBSDV) CP p10 [76], turnip mosaic virus (TuMV) 6K2 protein [77], and garlic virus X (GarVX) MP p11 [78], induced ER stress and the unfolded protein response (UPR), some of which could ultimately elicit a hypersensitive reaction (HR) in host plants. Plant virus MPs can modify the plasmodesmata (PDs), in favor of the virus movement, by replacing the desmotubule with expanded tubule structures [79,80], probably through interactions with plasmodesmata-located proteins (PDLPs) [80,81,82,83]. However, how the transportation of these organelle-specific localized proteins is affected by virus infection has not been studied. Thus, organelle-protoplast proteomics may be used to screen proteins with abnormal position upon virus infection. Since the chloroplast–protoplast proteomics method presented here is feasible for searching for the abnormal translocation of chloroplast proteins, the method could be applied extensively for research on nucleus-encoded proteins that can be specifically targeted into organelles other than chloroplast, which could also be influenced by pathogen infection.

### 3.4. Candidate for Plant Virus Resistance Improvement

GO enrichment analysis of the 66 candidate chloroplast proteins in response to RSV infection found three specific proteins which regulate the biological process of protein targeting into chloroplast. Their UniProt accessions are K4CSN4, K4CR23, and K4BXN9. In addition, we analyzed the protein levels of these three ChRPs in protoplasts after RSV infection, and found that they all showed a downregulation after RSV infection. The amino acid sequences of K4CSN4, K4CR23, and K4BXN9 were searched by BLAST, and it was shown that they had highest homology to chloroplast inner membrane translocon Tic110, chloroplast signal recognition particle 54 kDa protein (cpSRP54), and heat shock protein HSP90-5 in chloroplast (HSP90C), respectively. Many studies have pointed out that these three proteins (Tic110, cpSRP54, and HSP90C) play important roles in regulating plant growth [84,85], chloroplast structure [86], plant virus infection [8,87], and the process of protein targeting into chloroplast [88,89,90]. For instance, HSP90C is a chloroplast-localized member from a highly conserved subfamily of HSP90 molecular chaperones. It has been implicated to be involved in plant disease symptoms, photomorphogenesis, and the translocation of nuclear-encoded ChRPs to the chloroplasts [91,92]. For example, *Arabidopsis* HSP90C interacted with PsbO1, and functions in protein translocation into the thylakoid lumen [93]. Peach latent mosaic viroid (PLMVd) infection resulted in the silencing of *HSP90C* gene by an RNAi mechanism, which induced chlorosis along with growth suppression phenotypes [8,87]. HSP90C was shown to interact with import intermediates of nuclear-encoded ChRPs proteins, and the suppression of HSP90 ATP*ase* activity can inhibit the translocations of a variety of ChRP precursors across the inner envelope membrane, indicating that Hsp90C functions in the translocation of nuclear-encoded ChRPs to the chloroplasts [92]. Thus, these three key proteins (K4CSN4, K4CR23, and K4BXN9) may play essential roles in the interaction of chloroplast and be influenced by plant viruses for infection and symptom development. It is implied that they may serve as potential antiviral targets for genetic manipulation and still need further investigation.

## 4. Material and methods

### 4.1. Plant Materials and Virus Inoculation

*N. benthamiana* plants were grown in pots in a 25 °C growth room under a 16 h/8 h light/dark cycle. RSV was inoculated on *N. benthamiana* as previously described [94,95]. In brief, the RSV inoculates were prepared from the leaves of RSV-infected adult rice plants which were collected from Zhejiang province in China. The *N. benthamiana* plants were mechanically inoculated with RSV at the eighth leaf growth stage (50 days after sowing). In parallel, the experimental controls were mock-inoculated with homogenized leaf tissues without RSV. The symptoms and the occurrence of disease were observed and recorded on a daily basis. *N. benthamiana* plants with typical systemic symptoms were examined by reverse transcription polymerase chain reaction (RT-PCR), and used in the subsequent procedures.

### 4.2. Isolation of Protoplast and Chloroplast

Protoplast isolation was performed as previously described [96,97,98], with *w*/*v* modifications. In brief, about 2 g of *N. benthamiana* leaf tissues were digested with 12.5 mL enzyme mixture (1.5% (*w*/*v*) cellulase R-10, 0.5% (*w*/*v*) macerozyme R-10, 5 mM 2-morpholinoethanesulfonic acid (MES), 0.1% (*w*/*v*) BSA, 10 mM CaCl_2_, and 0.4 M mannitol, pH 5.8), and incubated at 28 °C for 5 h in the dark. The cells were collected from the interface of 0.4 M mannitol-MES and 0.55 M sucrose solution after centrifugation. The purified protoplasts were diluted appropriately and counted with a hemocytometer under a microscope for protoplast yield. The viability of protoplasts was determined by the FDA staining method, as described [99]. 

Chloroplasts from intact protoplasts were isolated as described previously [100], and we made some modifications here. In brief, the protoplast solution was centrifuged at 300 *g* for 6 min, and the pellets were resuspended in protoplast lysis buffer (0.3 M sorbitol, 20 mM tricine ± KOH (pH 8.4), 5 mM EDTA, 5 mM EGTA, 10 mM NaHCO_3_, and 0.1% BSA), then force-filtered through two-level 15 μm nylon mesh. The released chloroplasts were immediately purified on a 40%/80% Percoll gradient by centrifugation at 3000 *g* for 25 min at 4 °C, then the chloroplasts at the interface of 40%/80% Percoll were collected, washed, and then resuspended in chloroplast resuspension buffer (0.3 M sorbitol, 20 mM tricine-KOH (pH 7.6), 5 mM MgCl_2_, 2.5 mM EDTA). The intact chloroplasts were diluted appropriately and counted with a hemocytometer under a microscope for chloroplast yield.

### 4.3. Blue Native PAGE Electrophoresis

A gradient BN-PAGE was performed as previously described [101,102] with some *w*/*v* modifications. In brief, the chloroplasts were lysed in hypoosmotic buffer (50 mM HEPES, 2 mM MgCl_2_, 1 mM EDTA, 1 mM NaF, 1 mM PMSF (pH 7.5)) for 30 min at 4 °C, and then centrifuged for 10 min at 14,000 *g* at 4 °C for collection of chloroplast membranes. Chloroplast membranes were washed with washing buffer (330 mM sorbitol, 50 mM BisTris–HCl (pH 7.0)), and collected by centrifugation at 300 *g* for 2 min at 4 °C, then resuspended in 25BTH20G buffer (20% (*w*/*v*) glycerol, 25 mM BisTris–HCl (pH 7.0)). An equal volume of resuspension buffer containing 2% (*w*/*v*) *n*-dodecyl-β-d-maltoside (DDM) was added with continuous mixing, and the solubilization of membrane–protein complexes was performed by incubating on ice for 30 min. Insoluble materials were removed by centrifugation at 15,000 *g* for 10 min. The supernatant was mixed with 0.1 volumes of 5% (*w*/*v*) Serva blue G and loaded onto a 1.0 mm-thick 4–15% (*w*/*v*) acrylamide gradient gel (101 mm × 73 mm). Electrophoresis was performed at 4 °C.

### 4.4. Protein Extraction

Protein extraction of protoplasts and chloroplasts was performed using the methods described by Chatterjee et al. [103]. Briefly, each sample was ground in a mortar in the presence of liquid nitrogen and extracted with 3 mL of SDS buffer (30% sucrose, 2% SDS, 0.1 M Tris-Cl, 5% β-mercaptoethanol, and 1 mM phenyl methyl sulfonyl fluoride (PMSF) (pH 8.0)). The extracts were sonicated 6 times for 15 s at 60 amps. Following sonication, 3 mL of Tris phenol buffer was added to the mixture and vortexed for 10 min at 4 °C. This mixture was centrifuged at 8000 *g* for 10 min at 4 °C, and phenolic phase was collected and re-extracted with 3 mL SDS buffer and shaken for 3–10 min. The mixture was centrifuged once again as before using the same settings, then the phenolic phase was collected and precipitated overnight with 4 volumes of 0.1 M ammonium acetate in methanol at −20 °C. Protein precipitates were obtained by centrifugation at 10,000 *g* for 30 min at 4 °C, and then washed three times with cold 0.1 M ammonium acetate, and finally with cold 80% acetone (-20 °C). The pellets were dried and used for further analyses.

### 4.5. Protein Digestion

The proteins were dissolved in 8 M urea, 50 mM sodium bicarbonate, then incubated in a compact Thermomixer (Thermo Scientific, Waltham, MA, USA) with 20 mM Tris(2-carboxyethyl)phosphine (TECP) for 60 min at 30 °C and 30 mM iodoacetamide at 25 °C for 40 min in darkness. The samples were then diluted with 1.6 M urea, 50 mM sodium bicarbonate, and then subjected to trypsin digestion (Sigma, St. Louis, MO, USA) at 1:50 enzyme/substrate ratio. The reaction mixture was incubated at 37 °C for 16 h, and formic acid (FA) was included to a final concentration of 1% following digestion. Prior to LC–MS/MS analysis, the resulting peptides were desalted using a Strata X 33 µm Polymeric Reversed Phase column (Phenomenex, CA, USA) and resuspended in 0.1% FA. 

### 4.6. Mass Spectrometric Acquisition

Samples were analyzed using an Ultimate RSLC nano UHPLC system (Thermo Scientific, Waltham, MA, USA) coupled to a Q Exactive HF MS/MS (Thermo Scientific). For MS analysis, 1 μg of the samples was analyzed on an Acclaim PepMap C18 analytical column (2 μm, 75 μm id × 50 cm, Thermo Scientific) at 40 °C. The peptides were separated by a 103 min linear gradient from 4% to 16% ACN with 0.1% formic acid at 200 nL/min, followed by a linear increase to 25.6% ACN in 68 min, to 32% in 5 min, to 40% in 7 min, and to 72% ACN in 5min. For DDA acquisition, the full scan was performed between 400 and 1500 *m*/*z*. The automatic gain control target for the MS/MS scan was set to 2e5. Normalized collision energy was 27%. The HRM DIA method consisted of a survey scan at 60,000 resolution from 350 to 1200 *m*/*z* (automatic gain control target of 3e6 or 50 ms injection time). Then, 41 DIA windows were acquired at 30,000 resolution (automatic gain control target 1e6 and auto for injection time). Normalized collision energy was 30%, mass range from *m*/*z* 400 to 1000 with 41 isolation windows (29 windows of 14 *m*/*z*, followed by 5 windows of 25 *m*/*z*, 2 windows of 34.5 *m*/*z*, 5 windows of 40 *m*/*z*). The spectra were recorded in profile type. 

### 4.7. Mass Spectrometric Raw Data Analysis

The DIA data were analyzed with Spectronaut 10, a mass spectrometer vendor-independent software from Biognosys. The default settings were used for the Spectronaut search. Retention time prediction type was set to dynamic iRT (correction factor for window 1). Interference correction on MS2 level was enabled. The false discovery rate (FDR) was set to 1% at peptide level. The DDA spectra were analyzed with the MaxQuant Version 1.5.6.5 analysis software using default settings. The minimal peptide length was set to 7. Search criteria included carbamidomethylation of cysteine as a fixed modification, oxidation of methionine and acetyl (protein N-terminus) as variable modifications. The mass tolerance for the precursor was 4.5 ppm, and for the fragment ions, was 20 ppm. The DDA files were searched against the tomato UniProt Fasta database (state 2016.06.9, 33,952 entries), the RSV proteins (state 2017.01.06, 447 entries), and the Biognosys iRT peptide sequences (11 entries). The identifications were filtered to satisfy FDR of 1% on peptide and protein level. Spectral libraries were generated using spectral library generation in Spectronaut with DDA measurements.

## 5. Conclusions

RSV infection caused the malformation of chloroplast structure and the whole reduction of chloroplast membrane protein complexes in *N. benthamiana* plants. We applied protoplast–chloroplast proteomics and established special filters to screen the nucleus-encoded ChRPs which were not transported into chloroplasts during RSV infection, and obtained 66 candidate proteins. GO enrichment analysis of these 66 candidate proteins, together with the analysis of chloroplast transit peptides, indicated that RSV infection changed the location of 66 nucleus-encoded ChRPs from chloroplast. RSV infection was indicated to regulate the relevant multiple biological process of protein targeting into chloroplast through three key proteins (K4CSN4, K4CR23, and K4BXN9), which might contribute to the abnormal chloroplast structure and function. Our results indicate the process of chloroplast transportation of nuclear-encoded ChRPs is a new layer of plant–virus interaction. Our strategy can be applied for research related to more organelles influenced by plant pathogens. 

## Figures and Tables

**Table 1:** The candidate proteins which the process transported to chloroplasts were blocked by RSV infection.

UniProt AC	Protein Names	GO IDs
K4C8H3	Uncharacterized protein	GO:0003842; GO:0004028; GO:0004029; GO:0005739; GO:0008270; GO:0009507; GO:0009651; GO:0010133; GO:0050897; GO:0072593
Q711R1	Allene oxide cyclase	GO:0009507; GO:0046423
K4B2I9	Uncharacterized protein	GO:0005524; GO:0006457
K4BT58	Uncharacterized protein	GO:0005509; GO:0009534; GO:0009570; GO:0009654; GO:0015979; GO:0019898; GO:0031977
K4B778	Uncharacterized protein	GO:0005741; GO:0008308
A8UDS9	Copper/zinc superoxide dismutase	GO:0006801; GO:0046872; GO:0055114
A9CM21	Voltage-dependent anion channel	GO:0005741; GO:0008308
A0A075F1U0	Constitutive plastid-lipid associated protein	
K4C647	Uncharacterized protein	GO:0009570; GO:0009941; GO:0016491
K4CNE8	ATP-dependent Clp protease proteolytic subunit	GO:0000302; GO:0004252; GO:0009570; GO:0009941; GO:0010468
K4B858	Uncharacterized protein	GO:0005622
Q10712	Leucine aminopeptidase 1	GO:0004177; GO:0008235; GO:0009507; GO:0030145
K4B3J8	Uncharacterized protein	GO:0008836; GO:0009089
K4ASX2	Uncharacterized protein	GO:0005623; GO:0045454; GO:0051920
P37222	NADP-dependent malic enzyme	GO:0004471; GO:0004473; GO:0006108; GO:0008948; GO:0009507; GO:0046872; GO:0051287
A0A140G1U2	ATP-dependent Clp protease proteolytic subunit	GO:0004252; GO:0009536
K4AZT2	Uncharacterized protein	GO:0009579; GO:0016021
K4DC13	Uncharacterized protein	GO:0005524; GO:0005759; GO:0006986; GO:0009507; GO:0046872; GO:0046914; GO:0051082; GO:0051085; GO:0051087; GO:1901671
K4BXN9	Uncharacterized protein	GO:0005524; GO:0005739; GO:0005774; GO:0006457; GO:0009408; GO:0009414; GO:0009570; GO:0009651; GO:0009704; GO:0009941; GO:0010157; GO:0045037
K4D4L5	Uncharacterized protein	GO:0005886; GO:0016655
K4C841	Uncharacterized protein	GO:0008152; GO:0016597
K4BFT9	Uncharacterized protein	GO:0016021
K4B413	Uncharacterized protein	GO:0000302; GO:0004130; GO:0005739; GO:0005774; GO:0005777; GO:0009506; GO:0009507; GO:0009735; GO:0009941; GO:0016021; GO:0020037; GO:0034599; GO:0042744
K4BBZ1	Uncharacterized protein	
A7U630	Chloroplast methionine sulfoxide reductase B1	GO:0006979; GO:0030091; GO:0033743
K4CH79	Uncharacterized protein	GO:0003735; GO:0005840; GO:0006412; GO:0009570; GO:0009941; GO:0032544
Q8W3Z8	Plastidic phosphoglucomutase	GO:0005975; GO:0016868
K4CXW3	Glutamyl-tRNA (Gln) amidotransferase subunit A	GO:0004040; GO:0005524; GO:0005739; GO:0009570; GO:0030956; GO:0032543; GO:0050567; GO:0070681
K4BCV6	Uncharacterized protein	GO:0004222; GO:0006518; GO:0009507; GO:0046872
K4BCU7	Uncharacterized protein	GO:0004747; GO:0006014
K4CGD5	Uncharacterized protein	GO:0003743; GO:0003924; GO:0005525; GO:0005622; GO:0006412; GO:0009570; GO:0009941
K4BXX3	Alpha-1,4 glucan phosphorylase	GO:0005737; GO:0005980; GO:0008184; GO:0030170
K4BLP5	Uncharacterized protein	
E5LBC4	Prephenate aminotransferase	GO:0008483; GO:0009058; GO:0030170
Q6T7F1	Phosphoribosylaminoimidazole carboxylase	GO:0004638; GO:0005524; GO:0006189; GO:0046872
K4C5B9	Uroporphyrinogen decarboxylase	GO:0004853; GO:0005829; GO:0006782; GO:0006783; GO:0009570; GO:0009735; GO:0009941
K4BJL2	Uncharacterized protein	GO:0003735; GO:0005840; GO:0006412
K4CPX9	Uncharacterized protein	GO:0000027; GO:0003735; GO:0005762; GO:0006412; GO:0019843
K4ASZ2	Uncharacterized protein	GO:0003824; GO:0009058; GO:0030170
K4BDV0	Uncharacterized protein	GO:0000103; GO:0004020; GO:0004781
K4ASJ9	Uncharacterized protein	GO:0009535; GO:0009941
K4BEB0	Uncharacterized protein	GO:0000287; GO:0004614; GO:0005829; GO:0005978; GO:0006006; GO:0009570; GO:0009590; GO:0010319; GO:0019252; GO:0019388
K4B075	Uncharacterized protein	GO:0003723; GO:0004827; GO:0005524; GO:0005737; GO:0005739; GO:0006433; GO:0009553; GO:0009570; GO:0010109; GO:0017101; GO:0048316; GO:0048481
K4BX77	3-isopropylmalate dehydrogenase	GO:0000287; GO:0003862; GO:0009098; GO:0009570; GO:0051287
K4CJD3	Uncharacterized protein	
A8UDS7	ABA2	GO:0016491; GO:0071949
Q2MI78	30S ribosomal protein S18	GO:0003735; GO:0006412; GO:0009507; GO:0019843; GO:0022627
K4CR23	Uncharacterized protein	GO:0003924; GO:0005525; GO:0006614; GO:0008312; GO:0009570; GO:0080085
K4CNE7	Uncharacterized protein	GO:0009535; GO:0022891; GO:0055085
K4D5G2	Uncharacterized protein	GO:0000287; GO:0005737; GO:0009570; GO:0009941; GO:0016791
K4BU13	Uncharacterized protein	GO:0000166; GO:0003729; GO:0009570
K4BY59	Uncharacterized protein	GO:0003824; GO:0009058; GO:0030170
Q84RD7	ZIP	GO:0015979; GO:0015995; GO:0046872; GO:0048529
K4BPB0	Uncharacterized protein	GO:0003735; GO:0006412; GO:0015934; GO:0022626; GO:0042254
K4BXB1	Uncharacterized protein	GO:0003735; GO:0006412; GO:0009570; GO:0009735; GO:0009941; GO:0015934
K4CSN4	Uncharacterized protein	GO:0009658; GO:0009941; GO:0016021; GO:0045037
K4B2B2	Phospho-2-dehydro-3-deoxyheptonate aldolase	GO:0003849; GO:0009073; GO:0009507
A0A0H3U3I7	Geranylgeranyl pyrophosphate synthase 5	GO:0008299; GO:0016740
K4AXX7	Protein translocase subunit SecA	GO:0005524; GO:0006605; GO:0009658; GO:0009941; GO:0010109; GO:0015462; GO:0016020; GO:0017038
K4C9K5	Uncharacterized protein	GO:0006457; GO:0015031
Q0MW94	1-deoxy-d-xylulose-5-phosphate reductoisomerase	GO:0008299; GO:0016853; GO:0030604; GO:0046872; GO:0070402
K4BDU3	Uncharacterized protein	GO:0009055; GO:0051536
K9JIM2	Heat shock protein 101	GO:0005524
K4BVZ1	Uncharacterized protein	GO:0016021
A0A0K1D9E3	Granule-bound starch synthase I	
K4CHH4	Uncharacterized protein	GO:0006457; GO:0015031

**Table 2:** The three representative proteins which are involved in modulating the biological process of protein targeting into chloroplast, affected by RSV infection.

GO ID	GO Term	*p*-Value	UniProt Accession	Protein Name
GO:0045036	protein targeting to chloroplast	0.00242	K4CSN4 K4CR23 K4BXN9	Tic110 cpSRP54 HSP90C

## Supplementary Materials

Supplementary materials can be found at http://www.mdpi.com/1422-0067/20/2/253/s1.

## Abbreviations

AltMV	Alternanthera mosaic virus
AMV	alfalfa mosaic virus
BaMV	bamboo mosaic virus
BN-PAGE	blue native PAGE
BsMV	barley stripe mosaic virus
CB	Cajal bodies
CF1β	chloroplast β-ATP*ase*
ChRG	photosynthesis-related genes
ChRP	chloroplast-related protein
CIRV	carnation Italian ringspot virus
CMV	cucumber mosaic virus
CP	coat protein
CpGK	chloroplast phosphoglycerate kinase
cPGK	chloroplast phosphoglycerate kinase
cpSRP54	chloroplast signal recognition particle 54 kDa protein
dpi	days post-inoculation
ER	endoplasmic reticulum
FA	formic acid
FC	fold change
Fd I	ferredoxin I
FDR	false discovery rate
GarVX	garlic virus X
GO	gene ontology
GRV	groundnut rosette virus
HC-Pro	helper component-proteinase
HR	hypersensitive reaction
HSP90C	HSP90 in chloroplast
iTRAQ	isobaric tags for relative and absolute quantification
MES	2-morpholinoethanesulfonic acid
MNSV	melon necrotic spot carmovirus
MVBs	multivesicular bodies
OEC	oxygen evolving complex
PC	plastocyanin
PD	plasmodesma
PDLP	plasmodesmata-located protein
PLMVd	peach latent mosaic viroid
PMSF	phenyl methyl sulfonyl fluoride
PSI	photosystem I
PSII	photosystem II
PVY	potato virus Y
RbCL	RuBisCO large subunit
RbCS	RuBisCO small subunit
RBSDV	rice black-streak dwarf virus
RNAi	RNA interference
RSV	rice stripe virus
RT-PCR	reverse transcription PCR
RuBisCO	ribulose-1,5-bisphosphate carboxylase/oxygenase
TBSV	tomato bushy stunt virus
TECP	Tris(2-carboxyethyl)phosphine
TMV	tobacco mosaic virus
ToMV	tomato mosaic virus
TP	transit peptide
TuMV	turnip mosaic virus
UGPase	UDP-glucose pyrophosphorylase
WB	western blot
MPRO	protoplasts from mock-inoculated healthy leaves
RPRO	protoplasts from RSV-infected leaves
MCHL	chloroplasts isolated from protoplasts from mock-inoculated healthy leaves
RCHL	chloroplasts isolated from protoplasts from RSV-infected leaves
