# Supplementary material for: Characterization of Proteins Involved in Chloroplast Targeting Disturbed by Rice Stripe Virus by Novel Protoplast–Chloroplast Proteomics
Source: Int J Mol Sci. 2019 Jan 10;20(2):253. doi: 10.3390/ijms20020253 (PMC6358847; doi:10.3390/ijms20020253)

## Slide 1
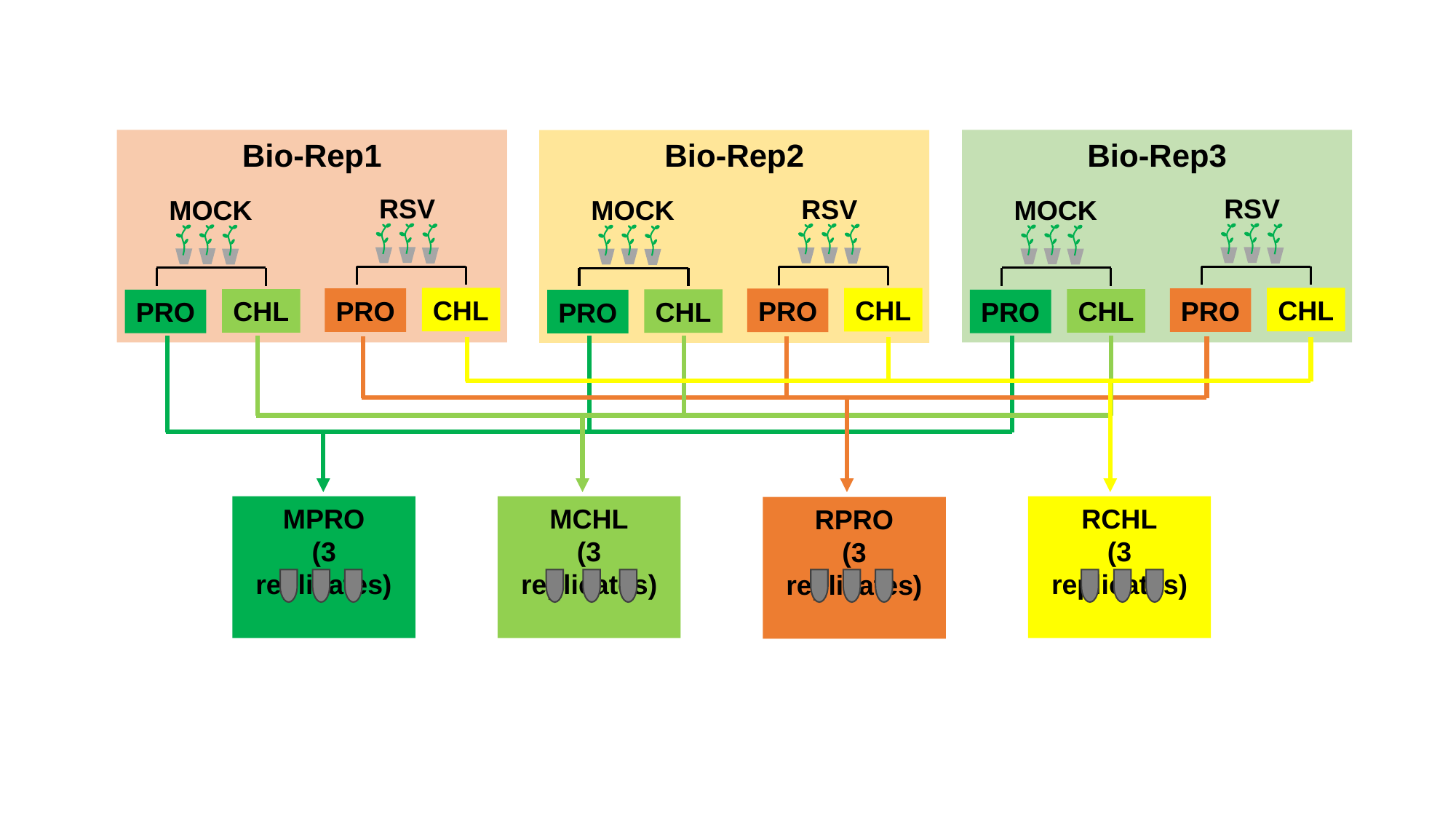

Bio-Rep1
RSV
MOCK
CHL
PRO
CHL
PRO
Bio-Rep3
RSV
MOCK
CHL
PRO
CHL
PRO
Bio-Rep2
RSV
MOCK
CHL
PRO
CHL
PRO
RCHL
(3 replicates)
MCHL
(3 replicates)
MPRO
(3 replicates)
RPRO
(3 replicates)

## Slide 2
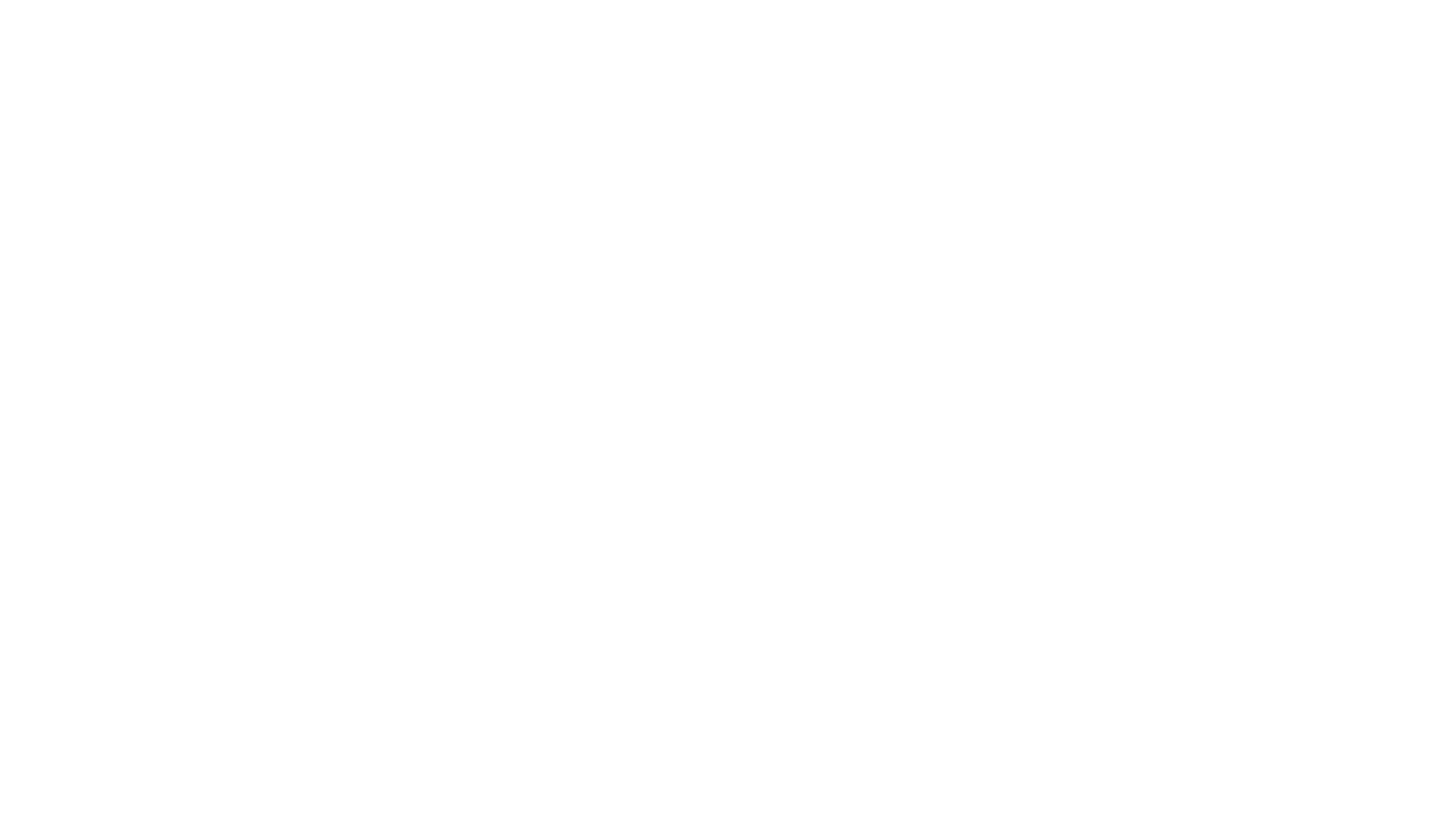

Supplement: Supplementary file 1 [file ijms-20-00253-s001.zip › Supplemental Figure S2.pptx]
