# Supplementary figures and images for: Characterization of Proteins Involved in Chloroplast Targeting Disturbed by Rice Stripe Virus by Novel Protoplast–Chloroplast Proteomics
Source: Int J Mol Sci. 2019 Jan 10;20(2):253. doi: 10.3390/ijms20020253 (PMC6358847; doi:10.3390/ijms20020253)

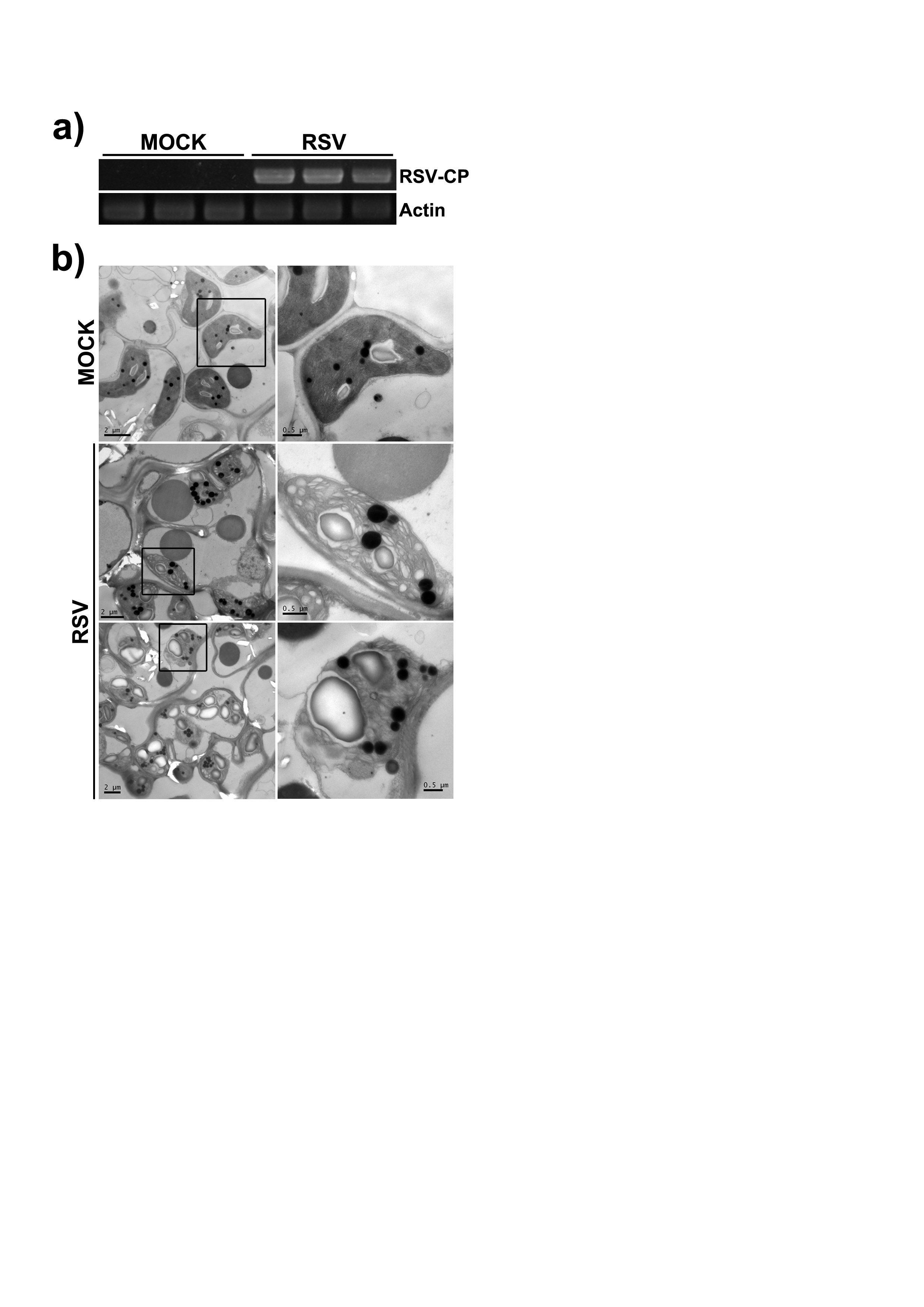

Supplement: Supplementary file 1 [file ijms-20-00253-s001.zip › Supplemental Figure S1.tif]

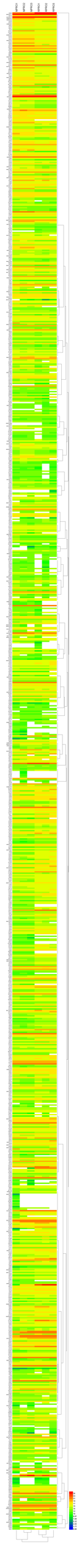

Supplement: Supplementary file 1 [file ijms-20-00253-s001.zip › Supplemental Figure S3.tiff]

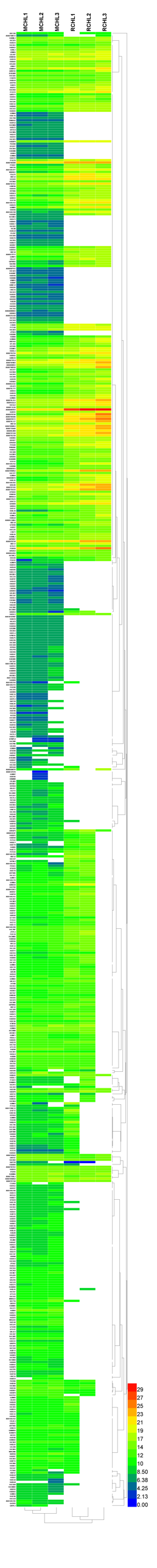

Supplement: Supplementary file 1 [file ijms-20-00253-s001.zip › Supplemental Figure S4.tiff]

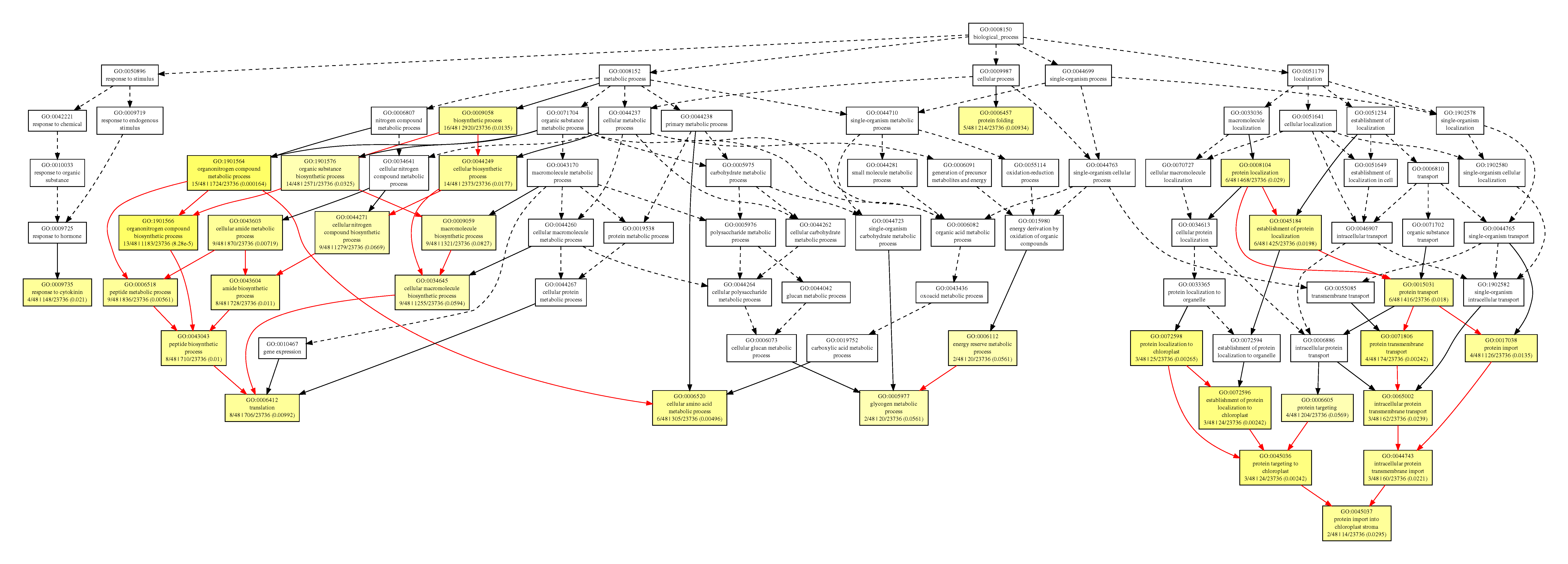

Supplement: Supplementary file 1 [file ijms-20-00253-s001.zip › Supplemental Figure S5.tif]

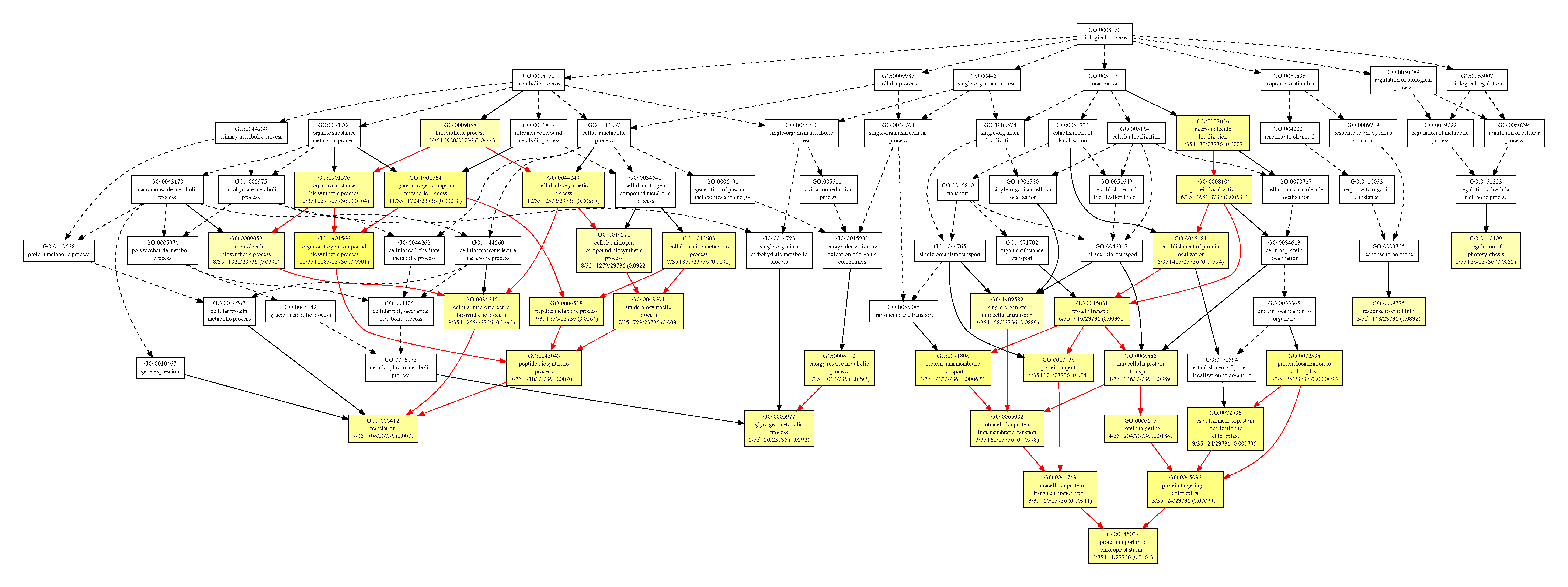

Supplement: Supplementary file 1 [file ijms-20-00253-s001.zip › Supplemental Figure S6.tif]
